# ST8SIA6-AS1 serves as a prognostic biomarker in cancer and exhibits oncogenic properties in prostate cancer

**DOI:** 10.3389/fgene.2025.1648077

**Published:** 2025-11-07

**Authors:** Yaoan Wen, Jiangbin Yang, Shuyuan Zhan, Yuanjing Huang, Guoqiang Chen, Song Zheng, Shaoxing Zhu

**Affiliations:** 1 Department of Urology, Fujian Medical University Union Hospital, Fuzhou, Fujian, China; 2 Department of Urology, The Second Hospital of Longyan, Longyan, Fujian, China

**Keywords:** ST8SIA6-AS1, prostate cancer, prognosis, meta-analysis, biomarker

## Abstract

**Background:**

The prognostic significance of long non-coding RNA ST8SIA6-AS1 remains ambiguous, and its biological role in prostate cancer (PCa) has not been thoroughly investigated. This study seeks to conduct a comprehensive meta-analysis to assess the clinical relevance of ST8SIA6-AS1 across different malignancies, with a particular emphasis on elucidating its functions in the advancement of PCa.

**Methods:**

Systematic searches were conducted in the PubMed, Embase, and Web of Science databases to select studies that met the criteria. Data, including hazard ratios (HR) with 95% confidence intervals (95% CI) and clinical pathological parameters, were collected. Subgroup analyses were performed based on sample size and tumor type. The expression profile analysis of PCa tissues was performed using the GTEx and TCGA databases. Changes in cell phenotype were assessed through Transwell migration assays and EdU proliferation assays. Additionally, the biological effects of ST8SIA6-AS1 were validated using an *in vivo* xenograft tumor model.

**Results:**

The meta-analysis including 11 studies with a total of 2,392 patients showed that high expression levels of ST8SIA6-AS1 are significantly positively correlated with reduced overall survival in patients with malignant tumors (HR = 1.48, 95% CI: 1.31–1.65), increased tumor size (OR = 2.07, 95% CI: 1.35–3.18), and advanced TNM staging (OR = 2.83, 95% CI: 1.77–4.52). Experimental findings confirmed that ST8SIA6-AS1 expression is significantly upregulated in PCa tissues and cells. Furthermore, the knockdown of ST8SIA6-AS1 inhibited cell migration, invasion, and proliferation *in vitro*, as well as suppresses cell growth *in vivo*.

**Conclusion:**

ST8SIA6-AS1, a significant oncogenic lncRNA, could promote disease progression in PCa and could serve as a significant prognostic marker for adverse outcomes in cancer.

## 1 Introduction

Cancer persists as a significant global public health issue, constituting the leading cause of disease-related mortality on an international level (Bray et al., 2021). Prostate cancer (PCa) represents a considerable clinical challenge among male malignancies, ranking as the second most commonly diagnosed cancer and the fifth leading cause of cancer-related deaths (Bray et al., 2024). According to the Global Cancer Statistics 2022, PCa was responsible for 1,466,680 newly diagnosed cases and 396,792 deaths globally (Bray et al., 2024). The elevated mortality rate, driven by a combination of unfavorable prognostic factors and swift disease progression, highlights the pressing clinical need for the development of innovative prognostic biomarkers.

Long non-coding RNAs (lncRNAs) represent a category of non-coding transcripts that exceed 200 nucleotides in length, are synthesized by RNA polymerase II, and do not possess the capacity to encode proteins (Statello et al., 2021). Recent research has revealed that, despite their lack of protein-coding ability, lncRNAs are integral to a variety of cellular and physiological functions. In the context of cancer biology, lncRNAs are implicated in the development of oncogenic characteristics by modulating essential malignant traits of tumor cells, such as proliferation, survival, metabolic reprogramming, and interactions with the tumor microenvironment (Statello et al., 2021; Bhan et al., 2017). This transformative understanding has spurred translational research aimed at investigating lncRNAs as potential diagnostic markers, therapeutic targets, and prognostic tools in oncology (Coan et al., 2024; Zhang XZ. et al., 2020). Among these lncRNAs, ST8 α-N-acetyl-neuraminide α-2,8-sialyltransferase six antisense RNA 1 (ST8SIA6-AS1), also referred to as APAL (Aurora A/Pololike-kinase 1-associated lncRNA), is situated on chromosome 10p12.33 and comprises three exons, spanning a total of 7,658 nucleotides (https://www.ncbi.nlm.nih.gov/gene/100506392). It has emerged as a significant oncogenic lncRNA in recent studies (Qiu et al., 2024). Accumulating evidence indicates that ST8SIA6-AS1 is aberrantly overexpressed in various solid tumors, exhibiting strong tumor-promoting effects in hepatocellular carcinoma (Zhang X. et al., 2020; Xue et al., 2023; Feng et al., 2023; Fei et al., 2020), cholangiocarcinoma (He et al., 2021), breast cancer (Fang et al., 2020; Luo et al., 2020; Chen et al., 2021; Wang et al., 2024), colorectal cancer (Wang et al., 2023), and lung cancer (Luo et al., 2020; Wang et al., 2023; Cao et al., 2020). Importantly, the abnormal expression levels of ST8SIA6-AS1 are significantly associated with clinicopathological parameters and patient prognosis across different malignancies. Nevertheless, the existing body of evidence is limited by constraints related to cohort size and discrepancies in mechanistic comprehension, which hinder the formation of a consensus concerning its clinical prognostic significance. In addition, the function of ST8SIA6-AS1 in the advancement of prostate cancer remains inadequately understood.

This research endeavor seeks to synthesize existing evidence through meta-analysis to quantitatively assess the relationship between ST8SIA6-AS1 expression levels and various clinicopathological characteristics, including TNM stage, tumor size, lymph node metastasis status, as well as survival outcomes in patients with tumors. Furthermore, we analyzed the expression differences of ST8SIA6-AS1 in prostate cancer tissues compared to adjacent normal tissues, as well as across different cell lines. We also evaluated the impact of ST8SIA6-AS1 knockdown on the malignant phenotype of prostate cancer cells, including their proliferation, migration, and invasion capabilities, to estimate its potential as a therapeutic target for prostate cancer.

## 2 Materials and methods

### 2.1 Search strategy

This study strictly adhered to PRISMA guidelines, with literature searches performed by two independent investigators across three databases: PubMed, Embase, and Web of Science (Moher et al., 2010). The search strategy was formulated as follows: “ST8SIA6-AS1” AND (“cancer” OR “tumor” OR “neoplasm” OR “carcinoma”), with a retrieval cutoff date of 31 May 2025. Simultaneously, we conducted a retrospective analysis of the literature by examining the reference lists of the selected studies and conducting manual screenings to identify further eligible publications.

### 2.2 Inclusion criteria and exclusion criteria

The criteria for literature selection were defined as follows: Inclusion Criteria: (1) Studies investigating the correlation between ST8SIA6-AS1 expression levels and clinicopathological parameters or prognostic indicators in patients with malignant tumors; (2) Provision of quantitative detection data for ST8SIA6-AS1 in human malignant solid tumor tissues; (3) Survival analyses must report hazard ratios (HR) with 95% confidence intervals (95% CI) or allow for reconstruction from survival curve data; (4) Publications must be restricted to English; (5) Explicit methodological description of ST8SIA6-AS1 detection techniques. Exclusion Criteria: (1) Duplicate publications (retaining the version with the most comprehensive data and largest sample size); (2) Non-primary research publications (such as reviews, case reports, conference abstracts, etc.); (3) Studies lacking extractable clinical outcome metrics; (4) Secondary analysis studies (e.g., systematic reviews or meta-analyses); (5) Research involving benign tumors or precancerous lesions; (6) Studies that include cohorts receiving therapeutic interventions (e.g., chemotherapy or targeted therapy). Two independent researchers performed the literature screening in strict adherence to the established criteria, with any discrepancies being resolved through arbitration by a third senior researcher.

### 2.3 Data extraction and quality assessment

Two investigators independently conducted data extraction and quality assessment following standardized protocols. Key variables were extracted from selected studies based on inclusion criteria, including the first author, publication year, patient cohort size, cancer type, detection methodology, HR, 95% CI for overall survival (OS) or progression-free survival (PFS), and recorded clinicopathological parameters. For studies that provided only Kaplan-Meier survival curves, Engauge Digitizer 4.1 software, along with spreadsheet calculations, was utilized for curve digitization and reconstruction of HR/CI (Wen et al., 2019). Study quality was assessed using the Newcastle-Ottawa Scale (NOS), with two independent investigators scoring each included study. Studies that achieved a total score of ≥6 were classified as high quality. Discrepancies in assessments were reconciled through discussion, with any unresolved controversies adjudicated by a third investigator to ensure objectivity (Wen Y. et al., 2018).

### 2.4 Bioinformatics analysis of ST8SIA6-AS1

Using GEPIA 2 (http://gepia2.cancer-pku.cn), which includes RNA sequencing data from 492 PCa tissues and 152 normal tissues sourced from the TCGA and GTEx databases, we analyzed the expression characteristics of ST8SIA6-AS1 in these samples. Additionally, we examined ST8SIA6-AS1 expression in a cohort of 49 paired tumor and adjacent non-cancerous tissue samples from the TCGA. The raw RNA-seq transcriptome data were log2 (x +1) transformed for normalization using R (version 4.0.2).

### 2.5 Cell cultures and transfection

The human prostate cancer cell line PC-3 used in this study was obtained from the American Type Culture Collection (ATCC). All cell lines were routinely cultured at 37 °C in a humidified incubator with 5% CO_2_. RWPE-1, LNCaP, PC-3, and 22Rv1 cells were maintained in RPMI 1640 medium, while DU 145 cells were cultured in high-glucose DMEM, both supplemented with 10% heat-inactivated fetal bovine serum (FBS; Gibco, Grand Island, NY, United States). To investigate the functional mechanisms of ST8SIA6-AS1, Lipofectamine 2000 transfection reagent (Invitrogen, United States) was employed to deliver ST8SIA6-AS1-specific siRNA (si-ST8SIA6-AS1) and scrambled negative control siRNA (si-NC; both synthesized by Invitrogen) into PC-3 cells. Transfection efficiency was evaluated 48 h post-transfection through qRT-PCR analysis of ST8SIA6-AS1 expression levels.

### 2.6 RNA extraction and qRT-PCR assays

RNA analysis was conducted following standardized molecular biology protocols. Total RNA was extracted from tissues and cultured cells using TRIzol reagent (Invitrogen). Reverse transcription was performed strictly according to the manufacturer’s instructions (PrimeScript RT Reagent Kit, TaKaRa) to generate complementary DNA (cDNA). Quantitative real-time polymerase chain reaction (PCR) was carried out using SYBR Premix Ex Taq reagents (TaKaRa), with thermal cycling parameters configured according to the manufacturer’s protocol. Glyceraldehyde-3-phosphate dehydrogenase (GAPDH) was used as the endogenous reference for normalizing ST8SIA6-AS1 expression levels.

### 2.7 Cell proliferation assay

Cell proliferation activity in prostate cancer cells was assessed using the KFluor 488 Click-iT EdU Imaging Detection Kit (KeyGen Biotech, Jiangsu, China). The experimental procedures were conducted as follows: Prostate cancer cells seeded in 24-well plates were treated with 50 μM EdU for 2 h, fixed with 4% formaldehyde, and subsequently incubated with the Click-iT reaction mixture to label EdU-positive cells. Nuclei were counterstained with Hoechst 33342 (blue fluorescence). Following image acquisition using an inverted fluorescence microscope, the proportion of EdU-positive cells (EdU-positive cells/total Hoechst-stained cells) was quantified using ImageJ software (National Institutes of Health, United States) to determine cellular proliferation rates.

### 2.8 Migration and invasion assays

Cell motility was quantitatively assessed using a Transwell chamber system (24-well plate, 8 μm pore size polycarbonate membrane; BD Biosciences) following a previously standardized protocol (Wen YA. et al., 2018). For invasion assays, the upper chambers were pre-coated with 0.33 mg/mL Matrigel matrix (Corning) for 2 h prior to experimentation to establish a basement membrane invasion model, while migration assays utilized uncoated membranes. Prostate cancer cells were resuspended in serum-free medium and seeded into the upper chambers at a density of 5 × 10^4^ cells per well, with the lower chambers filled with complete medium containing 10% FBS as a source of chemoattractant. After a 24-h incubation, non-migrated or non-invaded cells were removed from the upper chambers. The migrated or invaded cells on the lower membrane surface underwent methanol fixation followed by staining with 0.1% crystal violet for 15 min. Cell quantification was performed by counting the cells in five randomly selected microscopic fields under bright-field illumination, with the mean value serving as the quantitative metric.

### 2.9 Xenograft assays and shRNA treatment

A prostate cancer xenograft model was established using male BALB/c nude mice (4 weeks old, weighing 20–22 g; Experimental Animal Center of Fujian Medical University, Fuzhou, China). A stable knockdown of ST8SIA6-AS1 was achieved in PC-3 cells using lentiviral vectors containing shRNA specifically targeting ST8SIA6-AS1 (GenePharma, Shanghai, China). Subsequently, sh-ST8SIA6-AS1-transfected cells and control PC-3 cells were subcutaneously inoculated into the right flank of the mice (Wen YA. et al., 2018). Twenty-five days post-inoculation, euthanasia was performed via intraperitoneal injection of sodium pentobarbital (250 mg/kg), followed by dissection for tumor tissue collection and gravimetric analysis. All experimental procedures strictly adhered to protocols approved by the Institutional Animal Care and Use Committee of Fujian Medical University, ensuring compliance with the principles of the 3Rs (Replacement, Reduction, Refinement).

### 2.10 Statistical analysis

Meta-analysis was conducted using STATA 12.0 software (StataCorp LP, TX, United States) to calculate pooled effect sizes, including odds ratios (ORs) and hazard ratios (HRs) along with 95% confidence intervals (CIs). Publication bias was assessed using Begg’s rank correlation test, and sensitivity analysis was performed to validate the robustness of the results. Heterogeneity was evaluated using Cochran’s Q-test (significance threshold: p < 0.10) and quantified using the *I* ^2^ statistic. A fixed-effects model was applied when *I* ^2^ < 50% or Q-test p > 0.05; otherwise, a random-effects model was adopted. Continuous variable differences were analyzed using Student’s t-test, while categorical variables were compared via chi-square tests. All supplementary statistical analyses were conducted in SPSS 25.0 (IBM), with results expressed as mean ± standard error of the mean (S.E.M). Statistical significance was defined as two-tailed p < 0.05.

## 3 Results

### 3.1 Literature selection

**FIGURE 1:**
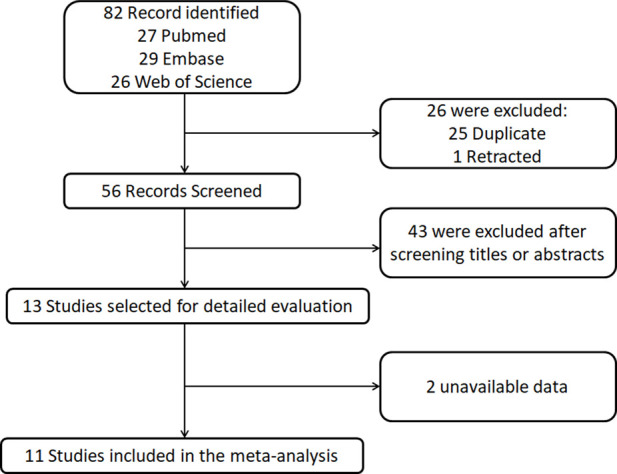
Flow diagram of the literature search and selection.

The literature screening process is illustrated in Figure 1. This study conducted a systematic search and screening in accordance with PRISMA guidelines. Initial searches across three databases (PubMed, Web of Science, and Embase) yielded 82 relevant articles, from which 25 duplicates and 1 retracted article were excluded through deduplication. Subsequent title and abstract screening of the remaining 56 articles resulted in the exclusion of 43 studies that did not meet the predefined inclusion criteria. Full-text evaluation of 13 potentially eligible articles further excluded 2 study due to critical data deficiencies. Following this rigorous screening process, 11 clinical studies that satisfied the methodological quality criteria were ultimately included for meta-analysis.

### 3.2 Characteristics of included studies

This meta-analysis ultimately incorporated 11 clinical studies (Table 1), encompassing a total of 2,392 patients with malignant tumors, covering the period from 2020 to 2024. It included distinct analyses from the same study Luo et al. (2020) for breast cancer (BC) and lung cancer (LC), as well as separate analyses from Wang et al. (2023) for LC and colorectal cancer (CRC). These distinct analyses were incorporated into their respective outcome assessments. All included studies extracted survival data based on Kaplan-Meier survival curves. The research covered five tumor types: liver cancer (*n =* 4), bile duct cancer (*n =* 1), breast cancer (*n =* 4), colorectal cancer (*n =* 1), and lung cancer (*n =* 3).

**TABLE 1:** Characteristic of included studies in this meta-analysis.

First author, year	Samples	Types of cancer	Outcome	Stage	Analysis method	Long intergenic non-coding ST8SIA6-AS1 expression		OS	
						high	low	HR	95% CI
Cao et al. (2020)	92	LUAD	OS	I-IV	Kaplan-Meier	46	46	2.85	1.11–7.32
Chen et al. (2021)	107	BC	OS	I-IV	Kaplan-Meier	54	53	1.49	1.15–2.23
Fang et al. (2020)	138	BC	OS RFS MFS	I-IV	Kaplan-Meier	82	56	3.37	1.29–8.77
Fei et al. (2020)	70	HCC	NA	NA	NA	35	35	NA	NA
Feng et al. (2023)	35	HCC	NA	NA	NA	18	17	NA	NA
He et al. (2021)	36	CHOL	OS	I-IV	Kaplan-Meier	18	18	1.5	0.35–6.38
Luo et al. (2020)	199	BC	RFS	I-IV	Kaplan-Meier	85	114	NA	NA
Luo et al. (2020)	64	LC	DFS	NA	Kaplan-Meier	NA	NA	NA	NA
Wang et al. (2023)	356	LC	OS	I-IV	Kaplan-Meier	NA	NA	1.41	1.2–1.66
Wang et al. (2023)	173	CRC	OS	NA	Kaplan-Meier	NA	NA	1.53	1.17–1.99
Wang et al. (2024)	1,068	BC	OS	I-IV	Kaplan-Meier	535	533	1.6	1.15–2.23
Xue et al. (2023)	60	HCC	OS	NA	Kaplan-Meier	NA	NA	1.535	1.0670–2.22
Zhang et al. (2020)	54	HCC	OS	I-IV	Kaplan-Meier	27	27	2.49	1.06–5.85

Abbreviations: LUAD, lung adenocarcinoma; BC, breast cancer; HCC, hepatocellular carcinoma, CHOL: cholangiocarcinoma; LC, lung cancer; CRC, colorectal cancer; OS, overall survival; RFS, Relapse-Free Survival; MFS, Metastasis-Free Survival; DFS, Disease-Free Survival; NA, not available.

### 3.3 Relationship between ST8SIA6-AS1 and prognosis

**FIGURE 2:**
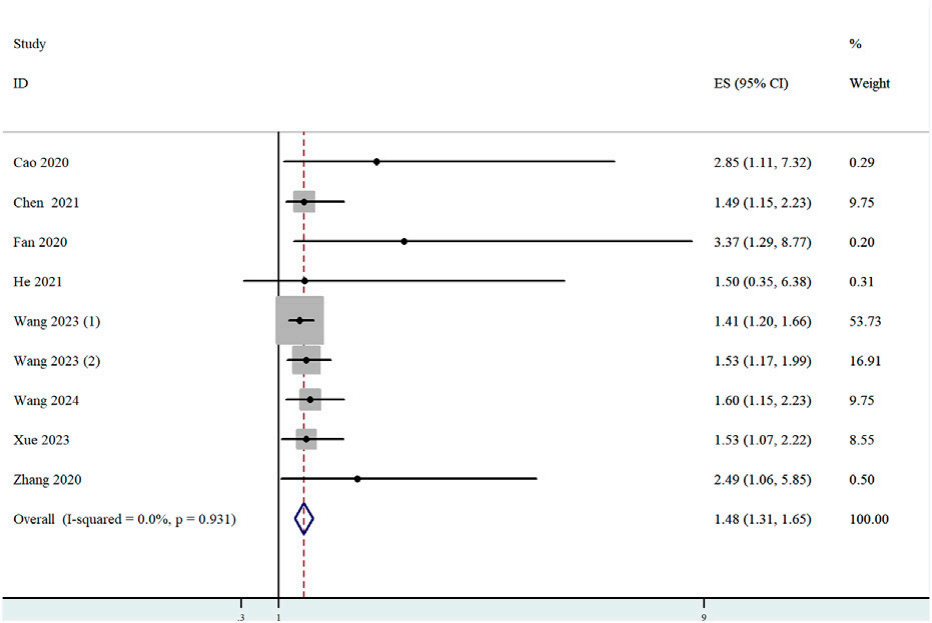
Forest plot for the relationship between ST8SIA6-AS1 expression and OS. Squares represented HR in each trial. The horizontal line crossing the square indicated the 95% CI.

Among the 11 studies that met the eligibility criteria, 8 studies (*n =* 2,024) reported analyses of the correlation between ST8SIA6-AS1 expression levels and OS. Heterogeneity testing revealed no significant statistical heterogeneity across the studies (*I* ^2^ = 0.0%, p = 0.931), confirming the consistency among results. A fixed-effects model was consequently employed to pool the effect sizes. The meta-analysis demonstrated that high ST8SIA6-AS1 expression was significantly associated with poorer OS (HR = 1.48, 95% CI: 1.31–1.65, p < 0.001; Figure 2).

**FIGURE 3:**
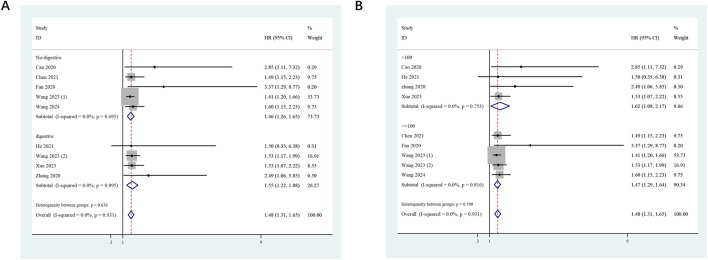
Forest plots evaluating the stratified analyses. Forest plots evaluating the stratified analyses of ST8SIA6-AS1 expression on OS in regard to subgroup including tumor type **(A)** and sample size **(B)**.

To investigate the potential relationship between ST8SIA6-AS1 and OS, stratified subgroup analyses were conducted based on tumor type and sample size. (1) In the digestive system tumor subgroup (HR = 1.55, 95% CI: 1.22–1.88, p < 0.001) and the non-digestive system tumor subgroup (HR = 1.46, 95% CI: 1.26–1.65, p < 0.001), high ST8SIA6-AS1 expression was significantly correlated with worse OS (Figure 3A). (2) When stratified by sample size, both large-sample studies (≥100 cases, HR = 1.47, 95% CI: 1.29–1.64, p < 0.001) and small-sample studies (<100 cases, HR = 1.62, 95% CI: 1.08–2.17, p < 0.001) revealed a significant relationship between ST8SIA6-AS1 and poor OS (Figure 3B). As well, no significant heterogeneity was detected among these subgroups.

### 3.4 Associations of ST8SIA6-AS1 expression with clinicopathological features

Aggregate analysis of clinicopathological parameters across included studies (Table 2) revealed a significant positive association between high ST8SIA6-AS1 expression and tumor malignant progression. Specifically: (1) TNM stage (*n =* 471): Higher expression levels of ST8SIA6-AS1 were associated with worse TNM staging (OR = 2.83, 95% CI: 1.77–4.52, p < 0.001). (2) Tumor size (*n =* 358): The high-expression cohort demonstrated significantly elevated risk for large tumor size (OR = 2.07, 95% CI: 1.35–3.18, p = 0.001). (3) Lymph node metastasis (*n =* 224): High-expression status showed borderline association with lymph node metastasis (OR = 2.44, 95% CI: 0.98–6.07, p = 0.055), warranting further validation.

**TABLE 2:** The associations of lncRNA ST8SIA6-AS1 expression with clinicopathological features.

Clinicopathological parameters	Studies (n)	No. of patients	ST8SIA6-AS1 expression		OR (95% CI)	p-value	Heterogeneity		Model
			High	Low			*I* ^2^ (%)	P-value	
Age (Older vs. Younger)	6	557	264	293	1.15 (0.76, 1.74)	0.505	0.0%	0.489	Fixed
TNM stage (III-IV vs. I-II)	4	471	226	245	2.83 (1.77, 4.52)	<0.001	37.4%	0.187	Fixed
Lymph node metastasis (Yes vs. No)	3	224	107	117	2.44 (0.98, 6.07)	0.055	60.20%	0.081	Random
Tumor size (large vs. small)	5	358	179	179	2.07 (1.35, 3.18)	0.001	0.0%	0.548	Fixed

### 3.5 Publication bias and sensitivity analyses

**FIGURE 4:**
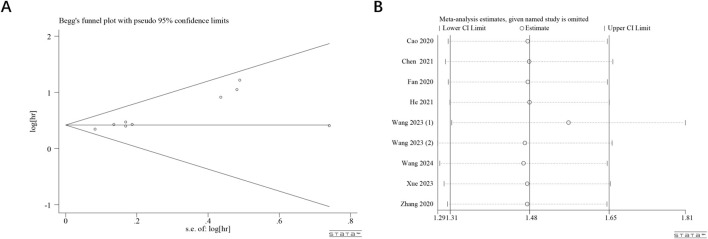
Publication bias and sensitivity analyses. Begg’s funnel plot **(A)** of potential publication bias and sensitivity analysis **(B)** for the meta-analysis among those studies reporting OS.

Publication bias in studies investigating the association between ST8SIA6-AS1 expression and OS was systematically assessed through Begg’s funnel plot (Figure 4A) along with rank correlation testing (z = 1.68, Pr > |z| = 0.093). The results suggested no significant publication bias. A sensitivity analysis was performed utilizing the leave-one-out method (Figure 4B). This analysis revealed that after excluding any individual study, the combined HR generally remained stable within the range of 1.31–1.65. This consistency suggests that the results are reliable.

### 3.6 ST8SIA6-AS1 was upregulated in PCa tissues and cells

**FIGURE 5:**
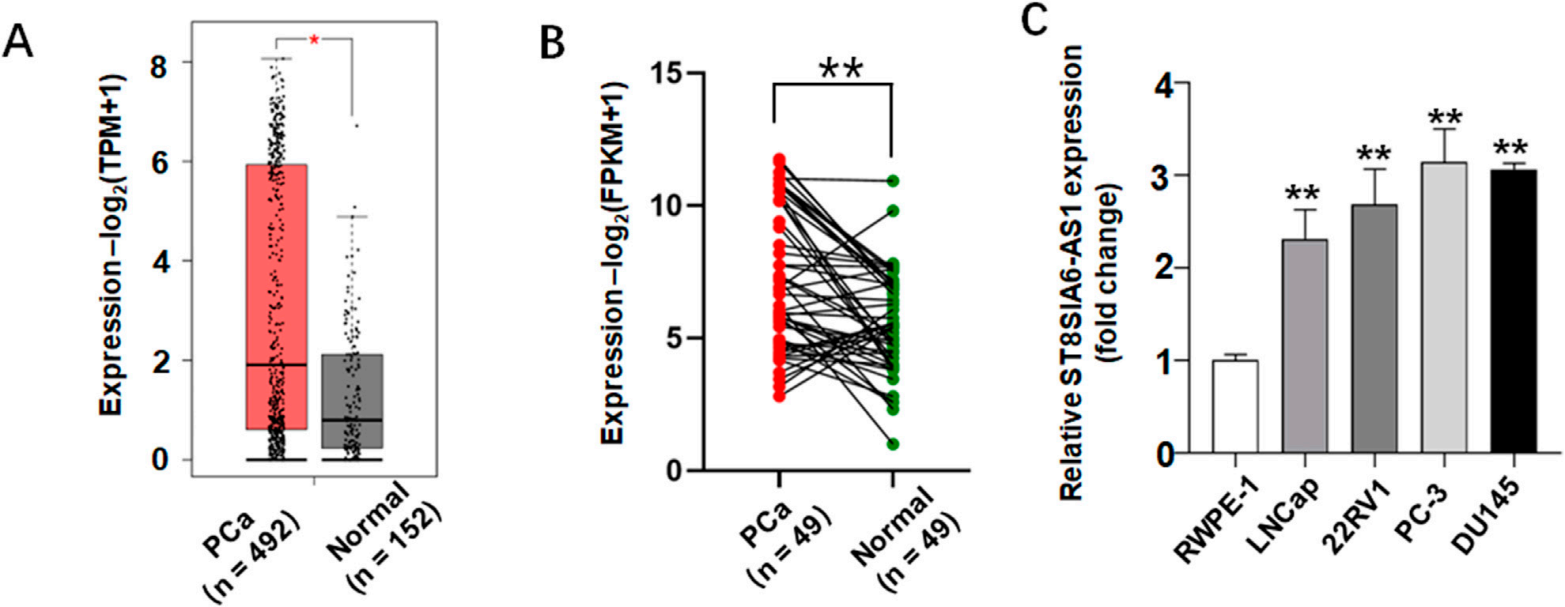
ST8SIA6-AS1 was significantly upregulated in prostate cancer (PCa). **(A)** Analysis via the GEPIA2 database demonstrates elevated ST8SIA6-AS1 expression in PCa tissues. **(B)** Comparison of ST8SIA6-AS1 expression levels between 49 PCa samples and matched adjacent normal tissues from TCGA using paired Student’s t-test. **(C)** Relative RNA expression of ST8SIA6-AS1 in normal human prostate epithelial cells (RWPE-1) and PCa cell lines (LNCaP, 22Rv1, PC-3, DU145), normalized to *GAPDH* (data presented as mean ± SEM, *n =* 3 independent experiments). *p < 0.05, **p < 0.01.

Transcriptomic data analysis based on the TCGA and GTEx databases revealed significantly differential expression patterns of ST8SIA6-AS1 in PCa tissues. Compared to normal prostate tissues, PCa samples exhibited a statistically significant upregulation of ST8SIA6-AS1 expression (Figures 5A,B). To further validate this finding, we measured expression profiles in four PCa cell lines (LNCap, 22Rv1, PC-3, and DU145) and normal human prostate epithelial cells (RWPE-1) using qRT-PCR. The findings indicated that the levels of ST8SIA6-AS1 were markedly increased in PCa cell lines in comparison to normal control samples, with the differences reaching statistical significance (Figure 5C).

### 3.7 ST8SIA6-AS1 knockdown inhibited cell proliferation, invasion and migration of PCa cells

**FIGURE 6:**
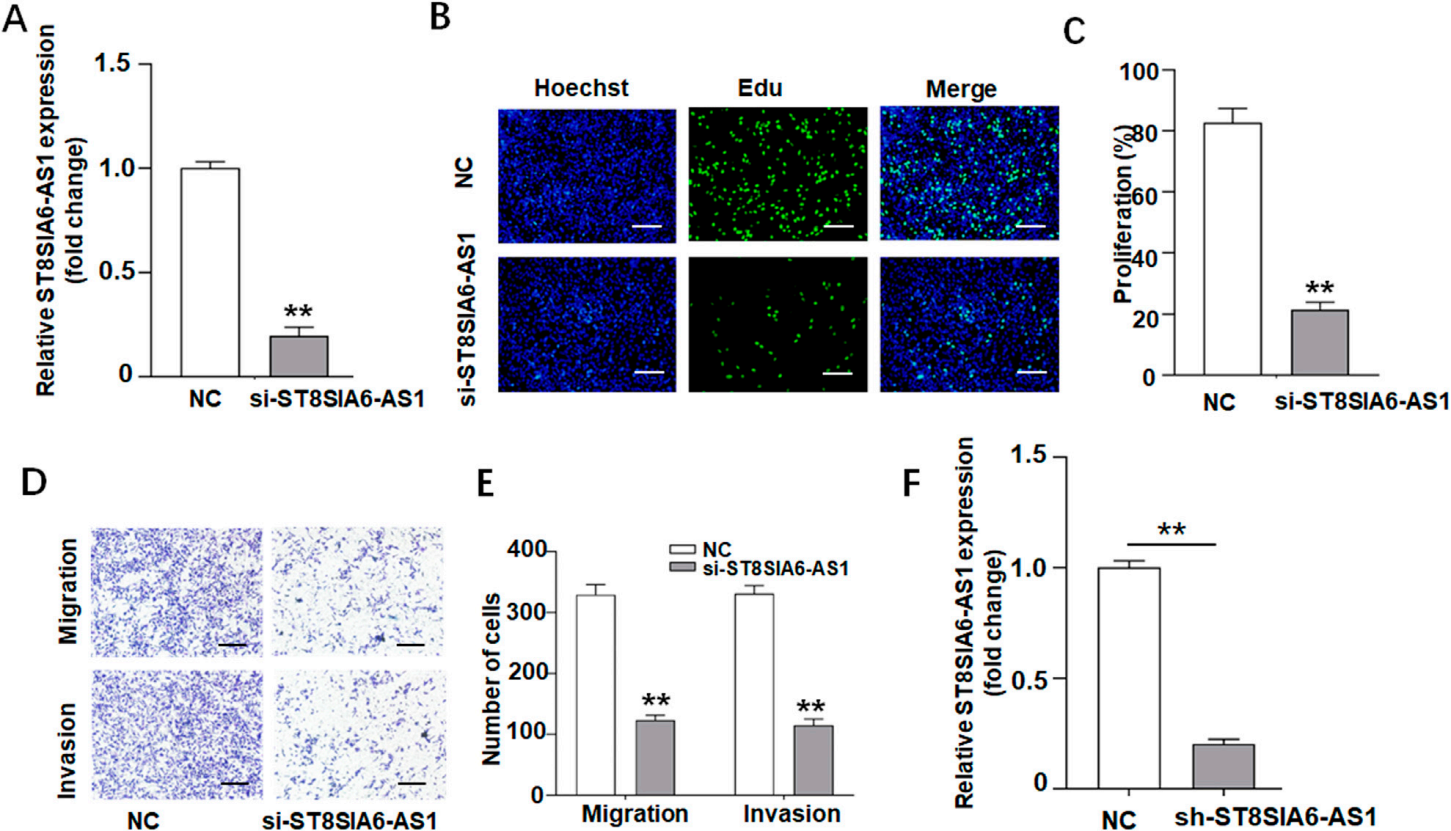
ST8SIA6-AS1 knockdown suppressed proliferation, invasion, and migration of PCa cells *in vitro*. **(A)** Silencing of ST8SIA6-AS1 expression in PC-3 cells via siRNA transfection. **(B,C)** EdU assay evaluating proliferative capacity after ST8SIA6-AS1 knockdown (Green: EdU-labeled proliferating nuclei; Blue: Hoechst-stained total nuclei). **(D,E)** Transwell migration and Matrigel invasion assays quantifying ST8SIA6-AS1 knockdown effects. **(F)** qRT-PCR validation of stable ST8SIA6-AS1 knockdown in PC-3 cells (data presented as mean ± SEM, *n =* 3 independent experiments). **p < 0.01.

Experiments utilizing gene silencing technology have demonstrated the critical role of ST8SIA6-AS1 in the malignancy of prostate cancer. Following shRNA-mediated specific knockdown, qRT-PCR confirmed an approximately 80% reduction in ST8SIA6-AS1 expression (Figure 6A), and demonstrated a significant downregulation of ST8SIA6-AS1 expression in sh-ST8SIA6-AS1 group compared to the sh-NC control group (Figure 6F). EdU proliferation assays revealed suggestively decreased EdU-positive rates in PC-3 cells after the knockdown of ST8SIA6-AS1 (Figures 6B,C). Transwell Matrigel invasion assays showed significantly fewer invasive and migratory cells in the experimental groups (Figures 6D,E).

**FIGURE 7:**
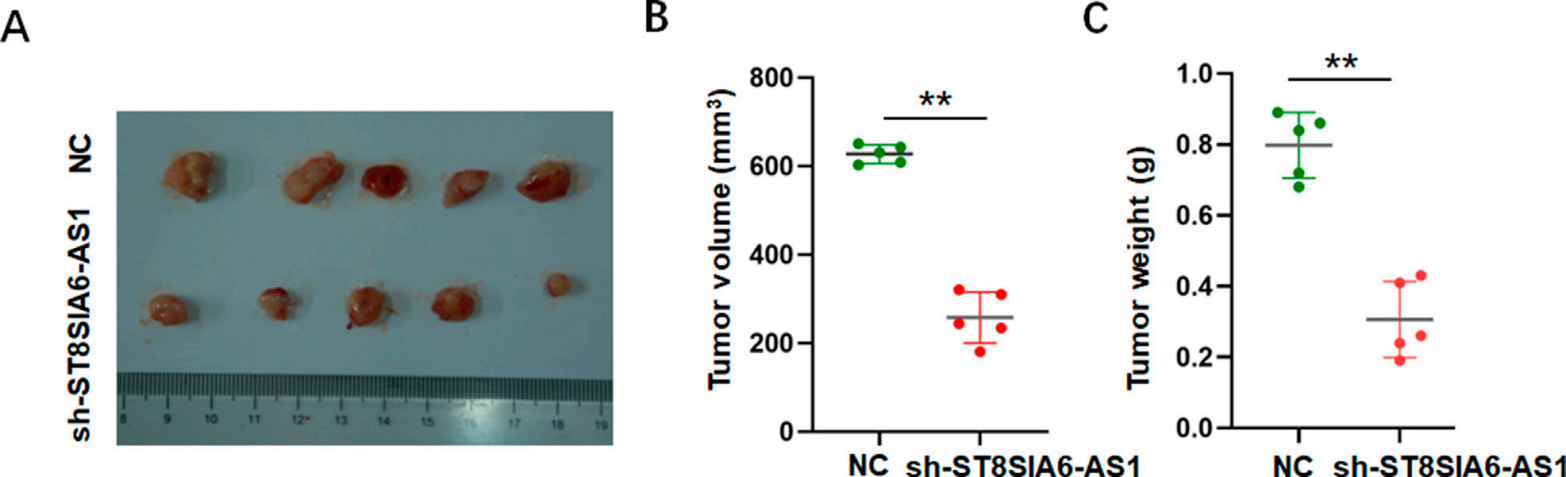
ST8SIA6-AS1 knockdown suppressed proliferation of PCa cells *in vivo*. **(A)** Representative tumor tissues harvested 25 days post-xenograft inoculation. **(B,C)** sh-ST8SIA6-AS1 significantly reduces tumor volume and weight. **p < 0.01.

To investigate the effects of ST8SIA6-AS1 knockdown *in vivo*, subcutaneous allograft tumor models were established by injecting PCa cells with stable ST8SIA6-AS1 knockdown. Compared to the control group, tumors in the sh-ST8SIA6-AS1 group exhibited significantly smaller and lighter (Figures 7A–C).

## 4 Discussion

ST8SIA6-AS1 has recently gained recognition as a significant oncogenic lncRNA, playing essential roles in tumorigenesis and showing promise as a novel biomarker and therapeutic target (Bhan et al., 2017; Coan et al., 2024; Qiu et al., 2024; Luo et al., 2020). Multiple studies have indicated that the expression of ST8SIA6-AS1 is associated with various clinicopathological features of tumors and patient prognosis. For instance, Feng et al. found that ST8SIA6-AS1 is significantly upregulated in HCC tissues and cell lines. Its elevated expression is positively correlated with serum alpha-fetoprotein (AFP) levels, lymph node metastasis, and TNM staging, while exhibiting a negative correlation with the overall survival rate of patients. Furthermore, the study revealed that ST8SIA6-AS1 promotes hepatocyte proliferation and invasion while inhibiting apoptosis by sponging miR-142-3p, thereby alleviating its suppression of HMGA1 (Feng et al., 2023). Similar findings were reported by Zhang and Fei et al. Zhang X. et al. (2020) and Fei et al. (2020), demonstrating that ST8SIA6-AS1 binds to specific miRNAs and functions as a competitive endogenous RNA (ceRNA) to regulate downstream gene expression, thus influencing tumor growth. Cao et al. found that ST8SIA6-AS1 is frequently overexpressed in LUAD cell lines, tissues, and plasma. Its elevated expression positively correlates with larger tumor size, lymph node metastasis, advanced TNM stage, and poor prognosis. Furthermore, functional experiments showed that ST8SIA6-AS1 exerts its effects by modulating the miR-125a-3p/NNMT axis; the alterations in cell viability and migration caused by its knockdown can be effectively reversed by miR-125a-3p silencing or NNMT overexpression (Cao et al., 2020). Wang and colleagues, through cell and animal model studies, suggested that ST8SIA6-AS1 promotes malignant proliferation of KRAS^G12C mutant cancers by activating the Aurora A/PLK1/c-Myc pathway. Knockdown of ST8SIA6-AS1 inhibits tumor cell proliferation and increases sensitivity to targeted therapies. Luo and others found through the analysis of multiple databases and self-collected samples that ST8SIA6-AS1 is overexpressed in various human cancers, including breast, lung, liver, kidney, and prostate cancers. This overexpression is linked to a poor clinical prognosis for patients, and high levels of ST8SIA6-AS1 expression correlating with higher tumor grades and stages. In patients with non-triple-negative breast cancer, as well as in those with breast and lung cancers, high ST8SIA6-AS1 expression serves as an independent predictor of recurrence and disease progression. Notably, concerning the molecular mechanism of ST8SIA6-AS1, Luo and colleagues propose that it can bind to PLK1 and Aurora A, enhancing Aurora A-mediated phosphorylation of PLK1. This interaction promotes PLK1 activation, thereby supporting the survival and proliferation of tumor cells (Luo et al., 2020). Although these studies focus on the specific mechanisms and associated genes of ST8SIA6-AS1 in different tumors, a consistent conclusion emerges: it is linked to poor prognosis across multiple tumor types as an oncogene. Additionally, it may promote cancer by adsorbing certain miRNAs, thereby regulating the expression of downstream genes, or by activating specific pathways, such as the Aurora A/PLK1 pathway. To establish a more reliable basis for the prognostic value of ST8SIA6-AS1, we conducted a meta-analysis to assess its clinical significance.

We constitute the inaugural meta-analysis aimed at systematically assessing the clinical relevance of ST8SIA6-AS1 across a range of malignancies. The aggregated data indicate a significant positive correlation between increased expression levels of this lncRNA and unfavorable clinical outcomes in cancer patients. Further stratified analyses suggest that ST8SIA6-AS1 may function as an independent prognostic biomarker for overall survival. Importantly, the expression levels of ST8SIA6-AS1 were found to be significantly associated with TNM stage and tumor size, and they also exhibited potential links to lymph node metastasis. Collectively, these findings implicate ST8SIA6-AS1 in the regulatory networks that govern tumorigenesis and progression, underscoring its translational potential as a clinical prognostic biomarker. Nevertheless, the existing evidence for prostate cancer remains inadequate, necessitating additional research to explore its expression patterns and biological functions within this particular malignancy.

This research, which employs an analysis of the TCGA and GTEx database alongside cellular experimental validation, represents the identification of the distinct overexpression profile of ST8SIA6-AS1 in prostate cancer cells and tissues. Targeted knockdown of ST8SIA6-AS1 expression via RNA interference technology resulted in a marked decrease in the migratory and invasive abilities of prostate cancer cells. These results corroborate previous studies, thereby reinforcing the role of ST8SIA6-AS1 as a pivotal regulator of tumor progression. Since this study is a retrospective analysis and does not involve *in vitro* or *in vivo* mechanistic experiments, the specific molecular mechanisms by which ST8SIA6-AS1 affects prostate cancer progression remain unclear. But, based on previous findings, we speculate that the possible mechanism by which ST8SIA6-AS1 influences prostate cancer progression involves sponging certain miRNAs, thereby regulating the expression of downstream genes, or activating specific pathways such as the Aurora A/PLK1 pathway to exert its oncogenic effects.

This meta-analysis underscores the clinical significance of ST8SIA6-AS1 across multiple malignancies; nevertheless, it is important to recognize several limitations associated with the study. Firstly, the predominance of studies conducted in China may limit the broader applicability of the findings. Secondly, the dependence on Kaplan-Meier survival curves for estimating effect sizes in studies that do not provide HRs and CIs may introduce potential biases during data extraction. In addition, due to limitations in the original research data, we cannot rule out the potential interference of collinearity between tumor size and TNM staging on the results, and the small sample sizes in certain subgroups may undermine the statistical power of the findings. Notably, this study presents a novel identification of an oncogenic role for ST8SIA6-AS1 in prostate cancer, demonstrating a significant positive correlation between its expression and both tumor invasiveness and metastatic potential. Nonetheless, the specific signaling pathways through which ST8SIA6-AS1 influences the progression of prostate cancer remain inadequately defined, indicating a need for further molecular mechanistic studies and functional validation experiments.

## 5 Conclusion

In conclusion, this research systematically establishes that the upregulation of ST8SIA6-AS1 expression is significantly correlated with tumor progression and unfavorable patient prognosis. Notably, prostate cancer models displayed a unique overexpression pattern of this lncRNA, and its genetic silencing effectively inhibited malignant characteristics, migratory ability, and invasive potential. Collectively, these results provide evidence that ST8SIA6-AS1 may facilitate prostate carcinogenesis by modulating pathways associated with tumor cell proliferation and metastasis. As a result, it could be regarded as a potential independent prognostic biomarker and a promising molecular target for the advancement of targeted therapeutic strategies.

## Data Availability

Meta-analysis summary statistics and wet-lab raw images are provided in the Supplementary Material or can be obtained from the corresponding author upon reasonable request. Gene-expression analyses were based exclusively on publicly available datasets (TCGA-PRAD via https://portal.gdc.cancer.gov and GEPIA2 http://gepia2.cancer-pku.cn); no new transcriptomic data were generated.
